# Supplementary material for: Increased power from conditional bacterial genome-wide association identifies macrolide resistance mutations in Neisseria gonorrhoeae
Source: Nat Commun. 2020 Oct 23;11:5374. doi: 10.1038/s41467-020-19250-6 (PMC7584619; doi:10.1038/s41467-020-19250-6)
Supplement: Supplementary file 1 — Supplementary Information [file 41467_2020_19250_MOESM1_ESM.pdf]

Increased power from conditional bacterial genome-wide association identifies macrolide resistance mutations in Neisseria gonorrhoeae (Ma et al. 2020)

Supplementary Information

Supplementary Tables 2
Supplementary Table 1 – Prevalence of RplD macrolide binding site mutations varies across datasets........ 2
Supplementary Table 2 – No substantial *in vitro* growth difference for the RplD G70D mutation in the 28BI laboratory strain background 3
Supplementary Table 3 – Variance in azithromycin MICs explained by known resistance and susceptibility genes. 4
Supplementary Figures 5
Supplementary Figure 1 – High genetic linkage between significant azithromycin MIC-associated variants in the GWAS 5
Supplementary Figure 2 – Distribution of r^2 values between significant variants (p-value < 2.97×10^{-7}) and 23S rRNA-associated unitigs in the single-locus GWAS 6
Supplementary Figure 3 – GWAS conditional on 23S rRNA mutations compared to unconditional GWAS results recovers similar results as in Figure 1 but does not control for dataset-specific confounders (*spr* and the intergenic region between genes WHO_F.2279 and 2280). 7
Supplementary Figure 4 – RplD G70 is part of the azithromycin binding pocket in the 50S ribosome from *Thermus thermophilus* (PDB ID: 4v7y). 8
Supplementary Figure 5 – Growth curve experiments for RplD G70D isogenic strains show no *in vitro* fitness cost 9
Supplementary References 10

Supplementary Tables

Dataset	Region and Timespan	Prevalence of RplD macrolide binding site mutations
Demczuk et al., 2015[1]	Canada 1989-2013	0.0439
Demczuk et al., 2016[2]	Canada 1982-2011	0.0804
Eyre et al., 2017[3]	Brighton, UK 2004-2011	0.0346
Ezewudo et al., 2015[4]	Global 1982-2011	0
Fifer et al., 2018[5]	UK 2004-2017	0.0200
Grad et al., 2016[6] and 2014[7]	US 2000-2013	0.0582
Harris et al., 2018[8]	Europe 2013	0.0258
Kwong et al., 2017[9]	Melbourne, Australia 2005-2014	0.0532
Lee et al., 2018[10]	New Zealand 2014-2015	0.0050
**Mortimer et al., 2020[11]**	**New York City 2011-2015**	**0.1014**
Ryan et al., 2018[12]	Ireland 2012-2016	0.0513
Sánchez-Busó et al., 2019[13]	Global 1979-2012	0.0212
**Yahara et al., 2018[14]**	**Kyoto and Osaka, Japan 1996-2015**	**0.1346**

Supplementary Table 1 – Prevalence of RplD macrolide binding site mutations varies across datasets. Datasets with prevalence over 10% are bolded.

	28BI	28BI RplD^{G70D} (E9)
**Best-fit values**		
logY0	3.285	3.822
k	0.3947	0.3879
Doubling Time	1.756	1.787
**95% CI (profile likelihood)**		
logY0	3.113 to 3.458	3.612 to 4.032
k	0.3725 to 0.4169	0.3610 to 0.4149
Doubling Time	1.663 to 1.861	1.671 to 1.920
**Goodness of Fit**		
Degrees of Freedom	16	16
R squared	0.9889	0.9831
Sum of Squares	0.3673	0.5431
Sy.x	0.1515	0.1842
**Number of points**		
# of X values	18	18
# Y values analyzed	18	18

Supplementary Table 2 – No substantial *in vitro* growth difference for the RplD G70D mutation in the 28BI laboratory strain background. Estimation of exponential phase growth parameters using GraphPad Prism following removal of lag phase data points and log-transformation of CFUs / mL; see Supplementary Figure 5 (bottom) for estimated best fit lines.

Mutation	Function	R^2 (lmg)	R^2 (first)	R^2 (last)
MtrR A39T	Pump upregulation	0.008703907	4.08E-05	0.014769
MtrR G45D	Pump upregulation	0.008911109	2.41E-03	0.015492
MtrR promoter	Pump upregulation	0.041388494	4.17E-02	0.037885
MtrR LOF	Pump upregulation	0.004493833	2.61E-04	0.0107
Mosaic Mtr	Pump upregulation and structural changes	0.076295392	4.20E-02	0.104465
23S rRNA A2059G	Ribosomal binding site modification	0.171732297	1.80E-01	0.167698
23S rRNA C611T	Ribosomal binding site modification	0.286807641	2.82E-01	0.290899
MtrC LOF	Pump loss-of-function (increased susceptibility)	0.056270124	4.85E-02	0.065024
RplD binding site mutations	Ribosomal binding site modification	0.010389559	1.62E-02	0.003228

Supplementary Table 3 – Variance in azithromycin MICs explained by known resistance and susceptibility genes. Variance explained by predictors was calculated using the relaimpo R package (version 2.2.3). The overall proportion of variance explained by the model was 66.5%. The variance explained by a predictor was determined by the change in model R^2 after inclusion of that predictor. Three approaches were used to calculate this change: the “first” metric compares a model without any predictors to a model with just the predictor of interest, the “last” metric compares a model with all predictors except the one of interest to a model with all predictors, and the “lmg” method averages the change in R^2 over all possible model subsets.

Supplementary Figures

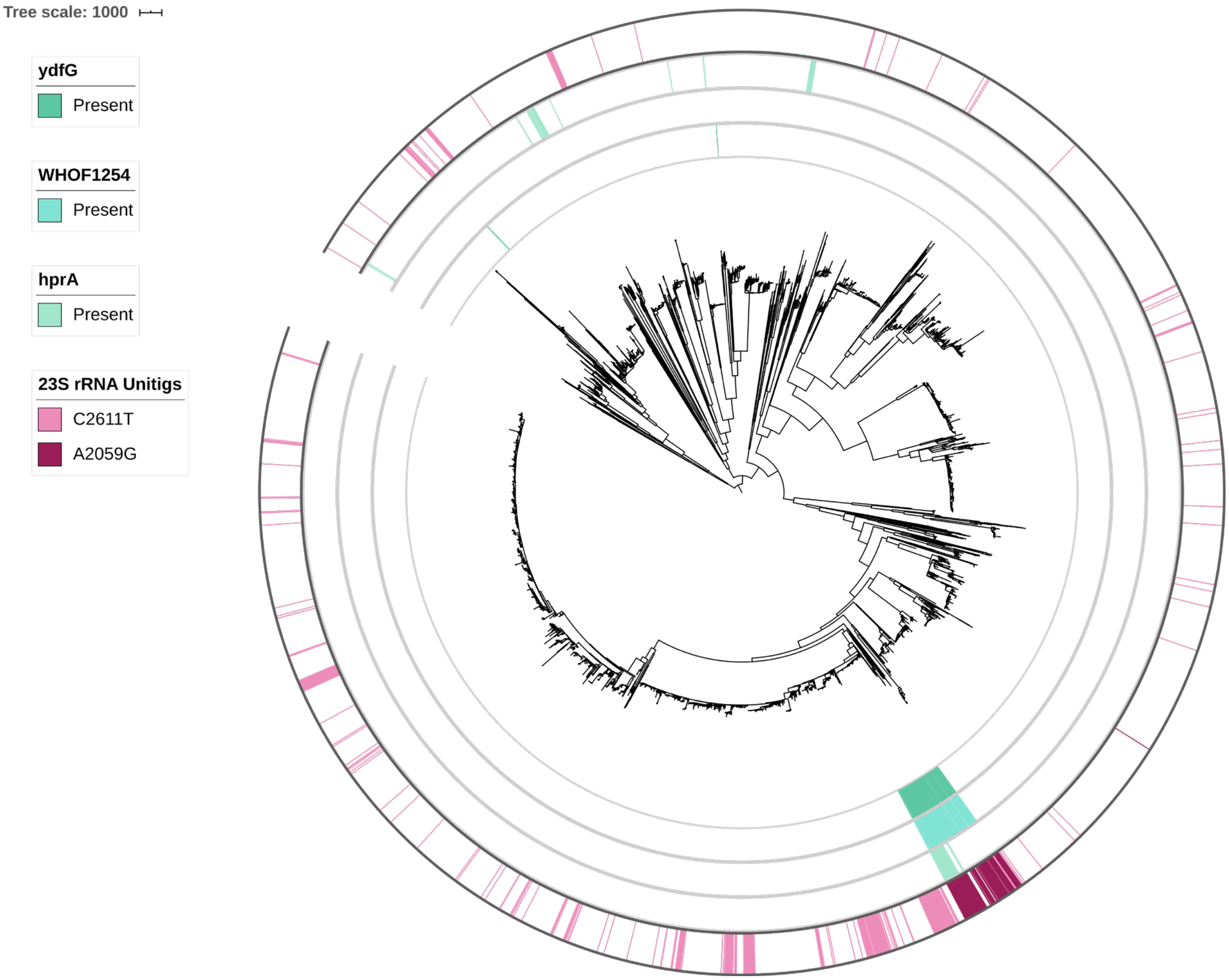

Supplementary Figure 1 – High genetic linkage between significant azithromycin MIC-associated variants in the GWAS. The recombination-corrected phylogeny from Figure 1 was annotated with the presence and absence of significant variants from the GWAS corresponding to 23S rRNA, *hprA*, WHO_F.1254, and *ydfG* (outermost to innermost). Branch length represents total number of substitutions after removal of predicted recombination.

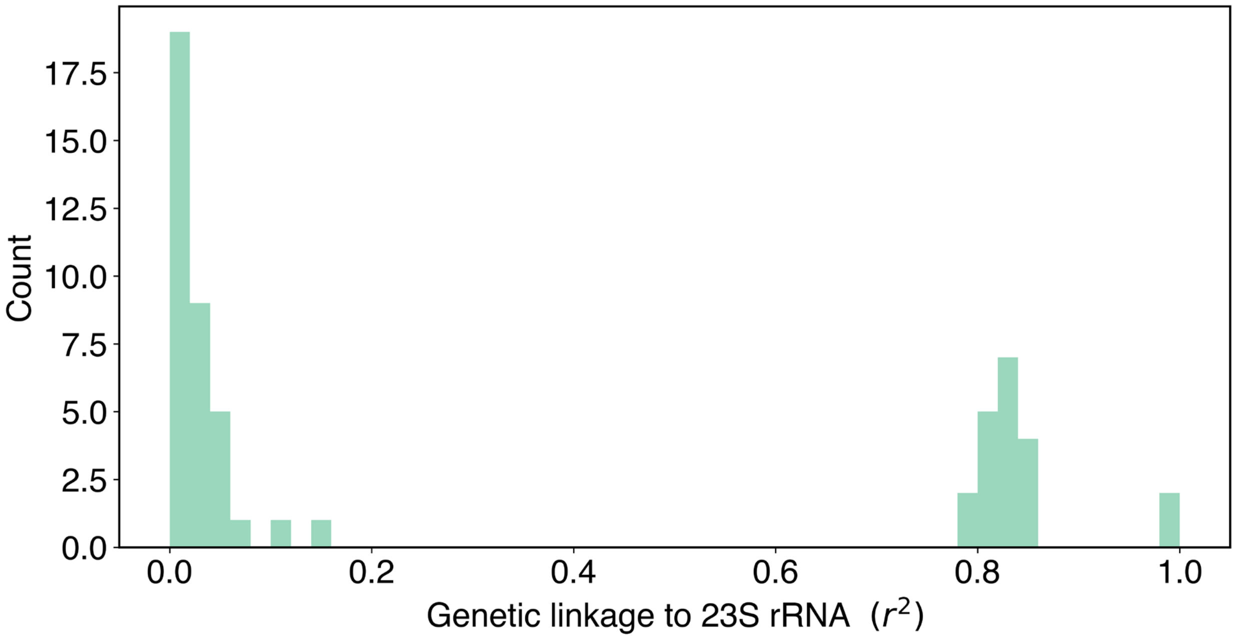

Supplementary Figure 2 – Distribution of r^2 values between significant variants (p-value < 2.97×10^{-7}) and 23S rRNA-associated unitigs in the single-locus GWAS. Significant variants with high linkage to 23S rRNA are likely to be spurious associations. See methods for details on calculation of r^2.

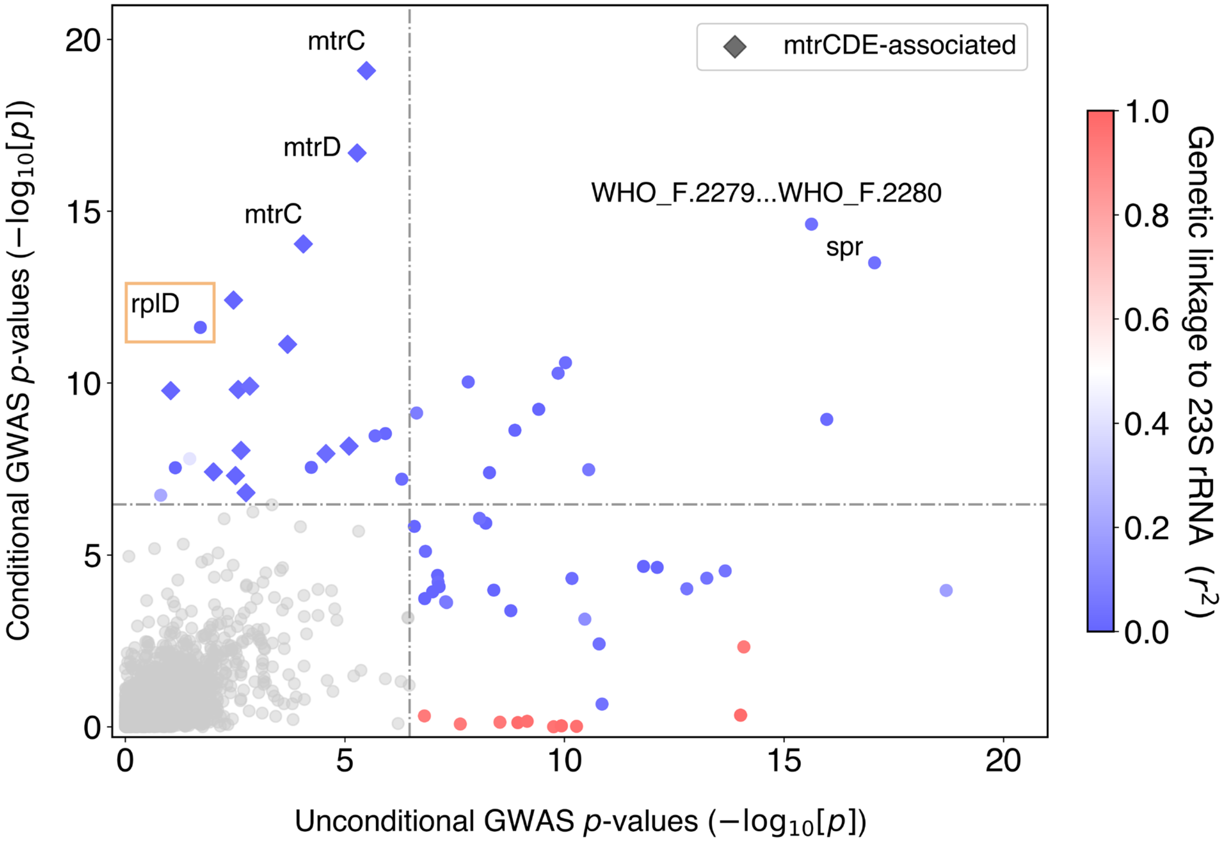

Supplementary Figure 3 – GWAS conditional on 23S rRNA mutations compared to unconditional GWAS results recovers similar results as in Figure 1 but does not control for dataset-specific confounders (*spr* and the intergenic region between genes WHO_F.2279 and 2280). As in Figure 1, genetic linkage measured by r^2 to 23S rRNA mutations A2059G and C2611T for significant variants is colored as indicated on the right. Variants associated with previously experimentally verified resistance mechanisms in the *mtrR* and *mtrCDE* promoters and coding regions are denoted in the legend. Bonferroni thresholds, calculated using the number of unique patterns, for both GWASes are depicted using a dashed line at 3.38×10^{-7}. Plot axes are limited to highlight variants associated with lower-level resistance; as a result, the highly significant 23S rRNA substitutions and *mtrC* indel mutations are not shown.

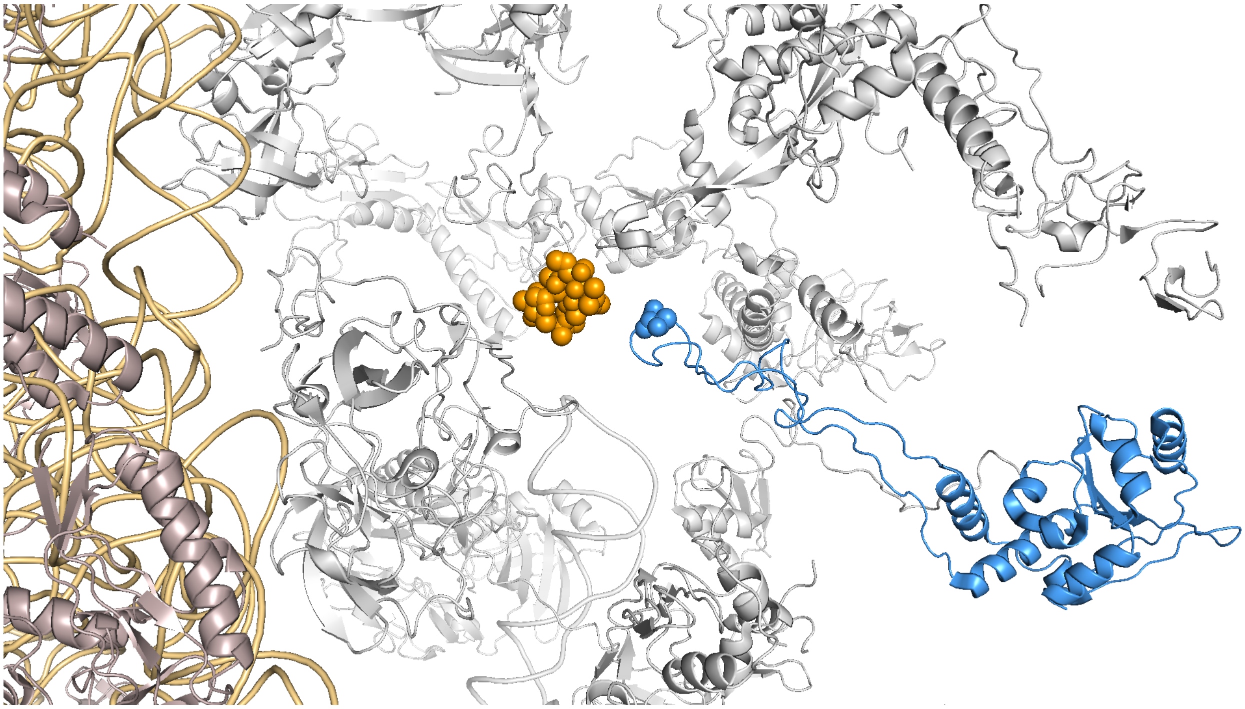

Supplementary Figure 4 – RplD G70 is part of the azithromycin binding pocket in the 50S ribosome from *Thermus thermophilus* (PDB ID: 4v7y). *N. gonorrhoeae* RplD is relatively similar to its *T. thermophilus* homolog (28.4% identical, 49.1% similarity using a BLOSUM62 matrix over 218 amino acids with 20 insertions/deletions). PyMOL (The PyMOL Molecular Graphics System, Version 2.0 Schrödinger, LLC) was used to depict azithromycin in orange and RplD in blue (with the G70 amino acid highlighted as blue spheres) and to hide the 23S rRNA for clarity.

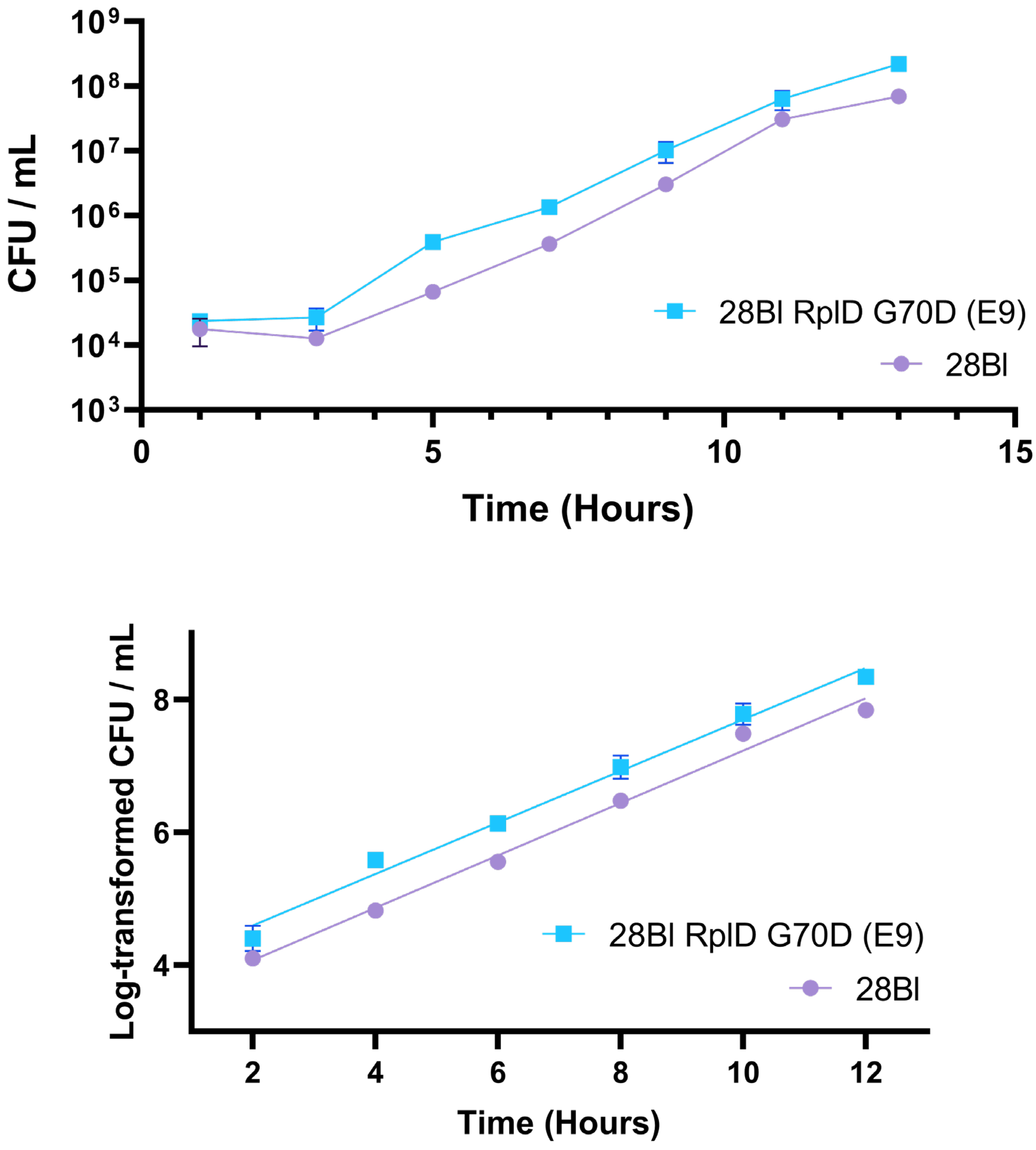

Supplementary Figure 5 – Growth curve experiments for RplD G70D isogenic strains show no *in vitro* fitness cost. Data are presented as mean values +/- SD calculated across the three technical replicates. Top – Calculated CFUs / mL from the full growth experiment for the two strains graphed on a logarithmic axis. Bottom – estimation of exponential phase best fit lines using GraphPad Prism following removal of lag phase data points and log-transformation of CFUs / mL; see Supplementary Table 2 for estimated parameters.

Supplementary References

1 Demczuk, W. *et al.* Whole-genome phylogenomic heterogeneity of Neisseria gonorrhoeae isolates with decreased cephalosporin susceptibility collected in Canada between 1989 and 2013. *J Clin Microbiol* **53**, 191-200, doi:10.1128/JCM.02589-14 (2015).

2 Demczuk, W. *et al.* Genomic Epidemiology and Molecular Resistance Mechanisms of Azithromycin-Resistant Neisseria gonorrhoeae in Canada from 1997 to 2014. *J Clin Microbiol* **54**, 1304-1313, doi:10.1128/JCM.03195-15 (2016).

3 Eyre, D. W. *et al.* WGS to predict antibiotic MICs for Neisseria gonorrhoeae. *J Antimicrob Chemother* **72**, 1937-1947, doi:10.1093/jac/dkx067 (2017).

4 Ezewudo, M. N. *et al.* Population structure of Neisseria gonorrhoeae based on whole genome data and its relationship with antibiotic resistance. *PeerJ* **3**, e806, doi:10.7717/peerj.806 (2015).

5 Fifer, H. *et al.* Sustained transmission of high-level azithromycin-resistant Neisseria gonorrhoeae in England: an observational study. *Lancet Infect Dis* **18**, 573-581, doi:10.1016/S1473-3099(18)30122-1 (2018).

6 Grad, Y. H. *et al.* Genomic Epidemiology of Gonococcal Resistance to Extended-Spectrum Cephalosporins, Macrolides, and Fluoroquinolones in the United States, 2000-2013. *J Infect Dis* **214**, 1579-1587, doi:10.1093/infdis/jiw420 (2016).

7 Grad, Y. H. *et al.* Genomic epidemiology of Neisseria gonorrhoeae with reduced susceptibility to cefixime in the USA: a retrospective observational study. *Lancet Infect Dis* **14**, 220-226, doi:10.1016/S1473-3099(13)70693-5 (2014).

8 Harris, S. R. *et al.* Public health surveillance of multidrug-resistant clones of Neisseria gonorrhoeae in Europe: a genomic survey. *Lancet Infect Dis* **18**, 758-768, doi:10.1016/S1473-3099(18)30225-1 (2018).

9 Kwong, J. C. *et al.* Whole-genome sequencing reveals transmission of gonococcal antibiotic resistance among men who have sex with men: an observational study. *Sex Transm Infect* **94**, 151-157, doi:10.1136/sextrans-2017-053287 (2018).

10 Lee, R. S. *et al.* Genomic epidemiology and antimicrobial resistance of Neisseria gonorrhoeae in New Zealand. *J Antimicrob Chemother* **73**, 353-364, doi:10.1093/jac/dkx405 (2018).

11 Mortimer, T. D. *et al.* The distribution and spread of susceptible and resistant *Neisseria gonorrhoeae* across demographic groups in a major metropolitan center. *medRxiv*, 2020.2004.2030.20086413, doi:10.1101/2020.04.30.20086413 (2020).

12 Ryan, L. *et al.* Antimicrobial resistance and molecular epidemiology using whole-genome sequencing of Neisseria gonorrhoeae in Ireland, 2014-2016: focus on extended-spectrum cephalosporins and azithromycin. *Eur J Clin Microbiol Infect Dis*, doi:10.1007/s10096-018-3296-5 (2018).

13 Sanchez-Buso, L. *et al.* The impact of antimicrobials on gonococcal evolution. *Nat Microbiol*, doi:10.1038/s41564-019-0501-y (2019).

14 Yahara, K. *et al.* Genomic surveillance of Neisseria gonorrhoeae to investigate the distribution and evolution of antimicrobial-resistance determinants and lineages. *Microb Genom* **4**, doi:10.1099/mgen.0.000205 (2018).
