# Supplementary material for: Increased power from conditional bacterial genome-wide association identifies macrolide resistance mutations in Neisseria gonorrhoeae
Source: Nat Commun. 2020 Oct 23;11:5374. doi: 10.1038/s41467-020-19250-6 (PMC7584619; doi:10.1038/s41467-020-19250-6)
Supplement: Supplementary file 3 — Description of Additional Supplementary Files [file 41467_2020_19250_MOESM3_ESM.pdf]

Descriptions of Additional Supplementary Files

File Name: Supplementary Data 1
Description: Annotated significant unitigs from GWAS conducted conditional on isolate country of origin.

File Name: Supplementary Data 2
Description: Annotated significant unitigs from GWAS conditional on isolate country of origin, dataset of origin, and number of 23S C2611T and A2059G rRNA mutations.

File Name: Supplementary Data 3
Description: Annotated significant unitigs from GWAS conditional on isolate country of origin and number of 23S C2611T and A2059G rRNA mutations.

File Name: Supplementary Data 4
Description: Multiple regression results for modeling log-transformed azithromycin MICs with baseline genetic covariates.

File Name: Supplementary Data 5
Description: Multiple regression results for modeling log-transformed azithromycin MICs with baseline genetic covariates and RplD mutations.

File Name: Supplementary Data 6
Description: SRA accession numbers, azithromycin resistance phenotypes and genotypes, assembly statistics, mapping statistics, and isolate metadata for all isolates in the N. gonorrhoeae global collection (n=4852).
