# Supplementary material for: Increased power from conditional bacterial genome-wide association identifies macrolide resistance mutations in Neisseria gonorrhoeae
Source: Nat Commun. 2020 Oct 23;11:5374. doi: 10.1038/s41467-020-19250-6 (PMC7584619; doi:10.1038/s41467-020-19250-6)
Supplement: Supplementary file 10 — Reporting Summary [file 41467_2020_19250_MOESM10_ESM.pdf]

nature research

Corresponding author(s): Yonatan Grad

Last updated by author(s): Sep 21, 2020

Reporting Summary

Nature Research wishes to improve the reproducibility of the work that we publish. This form provides structure for consistency and transparency in reporting. For further information on Nature Research policies, see Authors & Referees and the Editorial Policy Checklist .

Statistics

For all statistical analyses, confirm that the following items are present in the figure legend, table legend, main text, or Methods section.

n/a	Confirmed	
☐	☒	The exact sample size (*n*) for each experimental group/condition, given as a discrete number and unit of measurement
☐	☒	A statement on whether measurements were taken from distinct samples or whether the same sample was measured repeatedly
☐	☒	The statistical test(s) used AND whether they are one- or two-sided<br>*Only common tests should be described solely by name; describe more complex techniques in the Methods section.*
☐	☒	A description of all covariates tested
☐	☒	A description of any assumptions or corrections, such as tests of normality and adjustment for multiple comparisons
☐	☒	A full description of the statistical parameters including central tendency (e.g. means) or other basic estimates (e.g. regression coefficient) AND variation (e.g. standard deviation) or associated estimates of uncertainty (e.g. confidence intervals)
☐	☒	For null hypothesis testing, the test statistic (e.g. *F*, *t*, *r*) with confidence intervals, effect sizes, degrees of freedom and *P* value noted<br>*Give P values as exact values whenever suitable.*
☒	☐	For Bayesian analysis, information on the choice of priors and Markov chain Monte Carlo settings
☒	☐	For hierarchical and complex designs, identification of the appropriate level for tests and full reporting of outcomes
☐	☒	Estimates of effect sizes (e.g. Cohen's *d*, Pearson's *r*), indicating how they were calculated

Our web collection on statistics for biologists contains articles on many of the points above.

Software and code

Policy information about availability of computer code

Data collection	fastq-dump in SRA toolkit version 2.8.1 was used to acquire sequencing read data
Data analysis	BWA-MEM (version 0.7.17-r1188), Picard (version 2.8.0), BamQC (version 2.2.1), Pilon (version 1.16), SPAdes (version 3.12.0), Prokka (version 1.13), Roary (version 3.12), Gubbins (version 2.3.4), iTOL (version 4.4.2), BLASTn(version 2.6.0), MAFFT (version 7.450), FastBAPS (version 1.0.0), R (version 3.5.1), Pyseer (version 1.2.0), GATB (version 1.3.0), Python (version 3.6.5), Biopython (version 1.69), Geneious Prime (version 2019.2.1), Prism (version 8.2.0), custom code: https://github.com/gradlab/rplD-conditional-GWAS, PyMOL (The PyMOL Molecular Graphics System, Version 2.0 Schrödinger, LLC)

For manuscripts utilizing custom algorithms or software that are central to the research but not yet described in published literature, software must be made available to editors/reviewers. We strongly encourage code deposition in a community repository (e.g. GitHub). See the Nature Research guidelines for submitting code & software for further information.

Data

Policy information about availability of data

All manuscripts must include a data availability statement. This statement should provide the following information, where applicable:

- Accession codes, unique identifiers, or web links for publicly available datasets
- A list of figures that have associated raw data
- A description of any restrictions on data availability

In Supplementary Data 6, we have included accession numbers (via publicly hosted database NCBI SRA) for accessing all raw sequence data used for N. gonorrhoeae analyses, including all strains from the CDC GISP dataset. Intermediate outputs from the genomics pipeline (e.g., de novo assemblies) may also be available from the authors upon request. An interactive and downloadable version of the phylogeny and annotation rings used in Figure 2 and Supplementary Figure 1 is hosted at https://itol.embl.de/tree/1281032245351351597338246. Source data underlying all figures are available in the Supplementary Data or at https://github.com/gradlab/rplD-conditional-GWAS.

Additionally, we used the publicly available structure of the ribosome from Thermus thermophilus (PDB ID: 4v7y), and publicly available reference genomes available from NCBI (FA1090 - RefSeq accession: NC_002946.2, and NCCP11945 - RefSeq accession: NC_011035.1).

Field-specific reporting

Please select the one below that is the best fit for your research. If you are not sure, read the appropriate sections before making your selection.

☐ Life sciences ☐ Behavioural & social sciences ☒ Ecological, evolutionary & environmental sciences

For a reference copy of the document with all sections, see nature.com/documents/nr-reporting-summary-flat.pdf

Ecological, evolutionary & environmental sciences study design

All studies must disclose on these points even when the disclosure is negative.

Study description	Conditional genome-wide association study of Neisseria gonorrhoeae followed by experimental validation
Research sample	Genome sequencing data along with antimicrobial resistance phenotypes and resistance genotypes for n=4852 strains, obtained from a previous publication (Ma and Mortimer et al., 2020).
Sampling strategy	The sampling strategy used in that study: after literature search at the time of study initiation, all available studies with at least 10 isolates with antimicrobial resistance and whole-genome sequencing data were included in our meta-analysis global collection. This comprehensive sampling strategy gave us the largest number of collected gonococcal isolates to date.<br><br>For this study, all isolates with associated azithromycin MICs were used for GWAS / linear models (n=4505), and all 4852 isolates were used to characterize genomic diversity of RplD.
Data collection	Relevant studies were identified through a systematic literature review on Pubmed and Google Scholar by K.C.M. and T.D.M.
Timing and spatial scale	Dataset curation was conducted from August 2018 to January 2019 for the global meta-analysis collection, followed by computational and statistical analyses. These isolates span 65 countries collected over 38 years.
Data exclusions	Data were only excluded if they failed to meet pre-established genomics quality control benchmarks.
Reproducibility	Novel significant statistical associations with resistance (rplD) were experimentally validated in the laboratory. All attempts to repeat the transformation experiment (two biological replicates) were successful. **MIC E-tests were conducted once. Growth assays were conducted in triplicate (three technical replicates).** Significant associations with previously known resistance genes (23s rRNA, mtrC deletion, mtrCDE overexpression mutations, mtr mosaics) were used as computational "positive controls" to ensure method validity.
Randomization	Country and dataset of origin was a possible confounder for antibiotic resistance differences between isolates, and was included as a covariate in the GWAS to account for country-level differences in MIC testing and reporting practices and dataset-specific biases in sequencing.
Blinding	Blinding was not relevant to this study as it was an observational study without an interventional treatment.

Did the study involve field work? ☐ Yes ☒ No

Reporting for specific materials, systems and methods

We require information from authors about some types of materials, experimental systems and methods used in many studies. Here, indicate whether each material, system or method listed is relevant to your study. If you are not sure if a list item applies to your research, read the appropriate section before selecting a response.

Materials & experimental systems

n/a	Involved in the study
☒	☐ Antibodies
☒	☐ Eukaryotic cell lines
☒	☐ Palaeontology
☒	☐ Animals and other organisms
☒	☐ Human research participants
☒	☐ Clinical data

Methods

n/a	Involved in the study
☒	☐ ChIP-seq
☒	☐ Flow cytometry
☒	☐ MRI-based neuroimaging
